# P-185. Safety, Efficacy and Immunogenicity of Chikungunya Vaccines: A Living Systematic Review and Meta-Analysis

**DOI:** 10.1093/ofid/ofaf695.408

**Published:** 2026-01-11

**Authors:** Juan M Sambade, Jamile Ballivian, Mabel Berrueta, Agustin Ciapponi, Ariel Bardach, Agustina Mazzoni, Martin Brizuela, Noelia Castellana, Esteban Couto, Katharina Stegelmann, Julieta Caravario, Florencia Salva, Edward P K Parker, Xu Xiong, Andy Stergachis, Flor M Munoz, Pierre buekens

**Affiliations:** Instituto de Efectividad Clinica y Sanitaria IECS, Vicente López , Buenos Aires, Argentina; Instituto de Efectividad Clínica y Sanitaria, Buenos Aires, Ciudad Autonoma de Buenos Aires, Argentina; Instituto de Efectividad Clinica y Sanitaria, Buenos Aires, Ciudad Autonoma de Buenos Aires, Argentina; Instituo de Efectividad Clinica y Sanitaria, Buenos Aires, Ciudad Autonoma de Buenos Aires, Argentina; IECS, Ciudad Autónoma de Buenos Aires, Ciudad Autonoma de Buenos Aires, Argentina; Instituto de Efectividad Clinica y Sanitaria, Buenos Aires, Ciudad Autonoma de Buenos Aires, Argentina; Hospital General de Agudos Velez Sarsfield, Buenos Aires, Ciudad Autonoma de Buenos Aires, Argentina; Instituto de Efectividad Clinica y Sanitaria, Buenos Aires, Ciudad Autonoma de Buenos Aires, Argentina; Instituto de Efectividad Clínica y Sanitaria, Buenos Aires, Ciudad Autonoma de Buenos Aires, Argentina; Instituto de Efectividad Clinica y Sanitaria, Buenos Aires, Ciudad Autonoma de Buenos Aires, Argentina; Instituto de Efectividad Clinica y Sanitaria, Buenos Aires, Ciudad Autonoma de Buenos Aires, Argentina; Instituto de Efectividad Clìnica y Sanitaria, Buenos Aires, Ciudad Autonoma de Buenos Aires, Argentina; The London School of Hygiene & Tropical Medicine, London, England, United Kingdom; Tulane University, New Orleans, Louisiana; University of Washington, Seattle, Washington; Baylor College of Medicine Houston, Dallas, Texas; Tulane University, New Orleans, Louisiana

## Abstract

**Background:**

Chikungunya virus is a re-emerging global threat. Two vaccines—one live-attenuated and one virus-like particle (VLP)—have received approval. However, comparative safety and immunogenicity data across platforms and special populations remain limited.

**Methods:**

We conducted a living systematic review (LSR) and meta-analysis of clinical and preclinical studies evaluating the safety and immunogenicity of chikungunya vaccines. Databases were searched biweekly (2014–2025). Outcomes included adverse events, pregnancy outcomes, and immunogenicity (seroprotection, seroconversion), stratified by age group, vaccine platform (live-attenuated, VLP, mRNA, vector), and baseline serostatus. Meta-analyses used random-effects models; absolute and relative risks were calculated with 95% confidence intervals (CI).

**Results:**

We included 55 studies (18 clinical, 37 preclinical). Trials enrolled 8,279 vaccinated participants: 7,560 adults and 719 adolescents. Two studies reported 16 post-vaccination pregnancies, with five spontaneous abortions—three assessed as unrelated and two reported as unrelated serious adverse events in independent safety review. In seronegative adults, live-attenuated vaccines were associated with increased risk of arthralgia (RR 2.95, 95% CI 2.29–3.79), fever (RR 11.01, 95% CI 6.37–19.03), and headache (RR 2.07, 95% CI 1.78–2.40); similar trends were observed in adolescents. Seroprotection outcomes were reported as incremental proportions (IP) comparing vaccinated and placebo groups. In adolescents, IPs were 0.94 (95% CI 0.87–1.01) with VLA1553 and 0.68 (95% CI 0.59–0.76) with PXVX0317 at 30 days, increasing to 0.95 (95% CI 0.90–1.00) at 365 days. In adults, VLA1553 reached 0.99 (95% CI 0.97–1.01) at 30 days; PXVX0317 reached 0.86 (95% CI 0.81–0.91) at 30 days and 0.74 (95% CI 0.68–0.81) at 365 days; mRNA-1388 maintained 1.00 (95% CI 0.76–1.24) throughout. Seroconversion mirrored seroprotection.

**Conclusion:**

Chikungunya vaccines show high immunogenicity across platforms. Live-attenuated vaccines are more reactogenic in seronegative individuals. Data on pregnancy remains limited. Our LSR supports continued evidence synthesis and post-licensure safety monitoring.

**Disclosures:**

Flor M. Munoz, MD, Merck: Advisor/Consultant|Pfizer: Advisor/Consultant|Pfizer: Grant/Research Support

